# A Novel Pathogenic DNA Variation in the OCRL1 Gene in Lowe Syndrome

**DOI:** 10.4274/jcrpe.v3i1.06

**Published:** 2011-02-23

**Authors:** Enver Şimşek, Tülay Şimşek, Yıldız Dallar, Önder Can, Patrick J Willems

**Affiliations:** 1 Division of Pediatric Endocrinology, Department of Pediatrics, School of Medicine, Eskisehir Osmangazi University, Eskisehir, Turkey; 2 Department of Glaucoma, Ulucanlar Eye Research and Training Hospital, Ankara, Turkey; 3 Department of Pediatrics, Ankara Research and Training Hospital, Ankara, Turkey; 4 GENDIA Genetic Diagnostic Network, B-2020 Antwerp, Belgium; +90 505 496 23 02enversimsek06@hotmail.comDivision of Pediatric Endocrinology, Department of Pediatrics, Eskisehir Osmangazi University, School of Medicine, Eskiflehir, Turkey

**Keywords:** Oculocerebrorenal syndrome, novel pathogenic DNA variation

## Abstract

The oculocerebrorenal syndrome of Lowe (OCRL) is an X-linked disorder characterized by congenital cataracts, renal tubular dysfunction, cognitive problems and maladaptive behavior. The syndrome is caused by pathogenic DNA variations in the X-linked OCRL1 gene. A 24-month-old boy was referred to our hospital with delayed motor milestones, hypotonia, involuntary purposeless movements of hands and feet, congenital cataract, severe feeding difficulties, and failure to thrive. Physical examination at the age of 24 months revealed a body weight of 7350 g (-5.1 SDS). Length was 71 cm (-5.1 SDS) and head circumference 45 cm (-3.9 SDS). He had deep-set small eyes, frontal bossing, flat occiput, parietal prominence, bilateral congenital cataract, cryptorchid left testis, joint hypermobility, decreased muscle tone, and hyporeflexia. Biochemical analysis revealed the characteristic findings of renal Fanconi syndrome. Genetic analysis showed a novel pathogenic DNA variation (c.1528C>T) in exon 15 of the OCRL1 gene. Clinical findings and genetic analysis confirmed the diagnosis of OCRL syndrome.

**Conflict of interest:**None declared.

## INTRODUCTION

The oculocerebrorenal syndrome of Lowe (OCRL) was first recognized as a distinct disease in 1952 by Lowe et al (1). The diagnostic triad of OCRL or Lowe syndrome includes eye anomalies (congenital cataracts, infantile glaucoma) resulting in impaired vision, neurological deficits (infantile hypotonia with subsequent mental impairment, absent deep tendon reflexes), and renal tubular dysfunction of the Fanconi type with glomerulosclerosis resulting in progressive chronic renal failure and end-stage renal disease (2,3). The disease is caused by pathogenic DNA variations in the OCRL1 gene on chromosome Xq26.1, which encodes the phosphatidylinositol polyphosphate 5-phosphatase protein. This protein is primarily located in the trans-Golgi network, on endosomes, and at the endocytic clathrin-coated pits. It regulates the concentration of intracellular phosphatidylinositol (4,5) bisphosphate, which is a membrane phospholipid known to regulate many intracellular processes. More than 100 pathogenic DNA variations leading to Lowe syndrome have been described, of which more than 90% are located in 2 hot spots (exons 10-18 and 19-23) in the OCRL1 gene. 

We report here a novel pathogenic DNA variation in exon 15 of the OCRL1 gene in a patient with Lowe syndrome.

## CASE REPORTS

**Figure 1:**
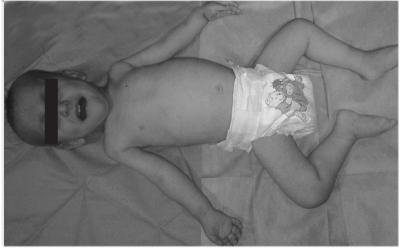
The clinical characteristics of the patient

A 24-month-old boy was referred to our hospital with delayed motor milestones, hypotonia, involuntary purposeless movements of hands and feet, congenital cataract, severe feeding difficulties, and failure to thrive. His past medical history revealed that he was born at term after a normal pregnancy period. He is the first child of a non-consanguineous marriage, and detailed pedigree analysis did not show any positive family history. Birth weight was 3600 g. Length at birth was 51 cm and head circumference 35.5 cm. The boy was on breastfeeding for only two months, and thereafter, on formula feeding for 8 months. At 24 months of age, he could not crawl or walk and could not sit without support. He was hospitalized several times to investigate the cause of his failure to thrive and recurrent respiratory tract infections, but there was no specific diagnosis to explain his symptoms and signs. Physical examination at age 24 months revealed a  body weight of 7350 g (-5.1 SDS). Length was 71 cm (-5.1 SDS) and head circumference 45 cm (-3.9 SDS). He had deep-set small eyes, frontal bossing, a flat occiput, parietal prominence, bilateral congenital cataract, cryptorchidism on the left side, joint hypermobility, decreased muscle tone, and hyporeflexia (Figure 1). The laboratory investigations are presented in Table 1. Serum thyroxine (T4) and free thyroxine (fT4) levels were low, while serum thyrotropin (TSH) level was in normal ranges. Radiographs of the wrists and knees demonstrated metaphyseal flaring, which is a characteristic sign of rickets. Renal ultrasonography showed evidence of nephrocalcinosis. The diagnosis of OCRL was based on the findings of congenital cataract, hypotonia, delayed motor developmental milestones, and renal Fanconi syndrome. Ophthalmological examination revealed bilateral congenital cataract and normal intraocular pressure. Cataract surgery was performed within the first month of hospital admission. For the management of his renal tubular acidosis, sodium citrate and potassium citrate (Polycitra) were administered. Neutral phosphate and calcitriol were administered to manage hypophosphatemic rickets. Following replacement therapy, serum TSH level was 3.7 μIU/mL and fT4 increased to 1.42 ng/dL.

To confirm the clinical diagnosis of Lowe syndrome, the OCRL1 gene was analyzed. Genomic DNA from the patient was used for polymerase chain reaction (PCR) amplification followed by direct sequencing of the entire coding region (exons 1-24) of the OCRL1 gene, including all intron-exon boundaries. A hemizygous c.1528C>T DNA variation was identified in exon 15 of the OCRL1  gene. This DNA variation alters a  glutamine into a premature stop codon on position 510 (p.Gln510X). The familial c.1528C>T mutation was also present in heterozygous state in the mother of the patient. Unfortunately, the mother did not undergo an ophthalmologic examination.

**Table 1:**
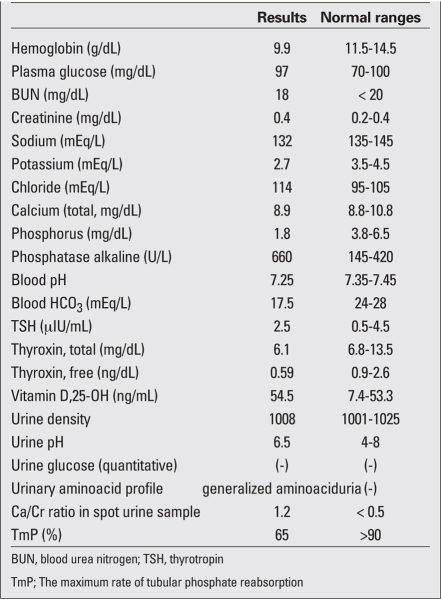
The laboratory results in the patient

## DISCUSSION

Patients with Lowe syndrome typically present in infancy with cataracts, progressive growth failure, hypotonia, and Fanconi syndrome, as seen in the present case. Because of the low prevalence of this disorder (1:200 000 -1:500 000 births) (4), clinicians may not be familiar with it. Lowe syndrome should be differentiated from other disorders associated with congenital cataract, hypotonia, proximal renal dysfunction and developmental delays, including congenital infections (such as TORCH infections), Zellweger syndrome,galactosemia, cystinosis, hereditary fructose intolerance and tyrosinemia. Clinical and laboratory studies eventually lead to the correct clinical diagnosis, which can be confirmed by molecular studies of the OCRL1 gene located on chromosome Xq24-26 (5,6,7). To date, more than 100 

pathogenic DNA variations have been reported in the OCRL1 gene (8). We present here an additional pathogenic DNA variation in the OCRL1 gene. The c.1528C>T mutation in exon 15 of the OCRL1 gene is a nonsense variation altering a glutamine into a premature stop codon on position 510 (p.Gln510X). This pathogenic DNA variation is predicted to result in a truncated OCRL1 protein or diminished OCRL1 mRNA due to mRNA decay. The c.1528C>T mutation is a novel pathogenic DNA variation not previously described in other patients with Lowe syndrome.

Proximal tubulopathy and OCRL1 gene mutation are also associated with Dent’s disease, a rare X-linked recessive inherited condition that affects the proximal renal tubules (9). The disorder has two different genetic backgrounds. Dent’s disease 1 is caused by mutations in the renal chloride channel CLCN5 that encodes a kidney-specific voltage gated chloride channel (10). Dent’s disease 2 is associated with OCRL1 gene mutation (11). Dent’s disease manifests itself through extreme thirst combined with dehydration, which leads to frequent urination, tubular proteinuria, hypercalciuria, nephrocalcinosis (kidney stones), aminoaciduria, phosphaturia, glycosuria, and impaired urinary acidification. However, this disorder does not present with congenital cataract and severe hypotonia. Cataracts are a hallmark of Lowe syndrome and are always present at birth. Congenital cataract, hypotonia, and the characteristic findings of proximal tubulopathy revealed the diagnosis of Lowe  syndrome in our patient, and the genetic analysis confirmed the diagnosis. Lowe syndrome cells have an elevated concentration of phosphatidylinositol 4,5-bisphosphate, the substrate for the OCRL1 protein (12). The presence of a reproducible cellular abnormality of the actin cytoskeleton in fibroblasts from patients with Lowe syndrome has been demonstrated (13). Actin polymerization plays a major role in the formation, maintenance, and proper function of adherens and tight junctions that have been shown to be crucial for the function of renal proximal tubule and for the lens differentiation.

The initial thyroid function tests in our case could be explained either by a state of  central hypothyroidism or by the development  of euthyroid sick syndrome, a condition often encountered in patients in intensive care units and thought to be due to starvation or critical illness (14). Our patient had renal tubular acidosis and chronic protein energy malnutrition, which we initially tried to correct. He was followed-up at three-monthly intervals. At the second follow-up visit, six month after initiation of replacement therapy, his mother reported that he had head control and had started crawling. Biochemical analysis revealed blood gases within normal ranges. Thyroid function tests had also improved following replacement therapy. This progress indicates that the initial thyroid dysfunction could be due to euthyroid sick syndrome. 

Genetic counselling should be done for carrier detection and prenatal testing. Molecular analysis of the OCRL1 gene is a more specific way to diagnose female carriers if the mutation in the proband is known (4). Genetic mutations should be documented first in the proband. As many as 94% of female carriers may be detected during ophthalmologic examination by slit-lamp biomicroscopy and the typical findings are multiple punctuate lenticular opacities or a single dense posterior cataract (15,16). However, even if the mother has normal ocular findings, mutation analysis should still be done due to the fact that though rarely, some women are nonpenetrant carriers and do not have the characteristic eye manifestations. Unfortunately, ophthalmic examination was not performed on the mother of the present case. The OCRL1 gene mutation analysis revealed that the familial c.1528C>T variation was also present in heterozygous state in the mother. If the mother of the proband is a carrier, as in this study, each male offspring has a 50% chance of being affected, while each female has a 50% chance of being a carrier. On the other hand, if the mother does not have the characteristic ocular findings and no OCRL1 mutations are found, then the mutation may be new and has a low recurrence risk (16). If genetic mutation is documented in the proband, prenatal testing is also possible for at-risk pregnancies, using either molecular analysis or biochemical testing (17).

In conclusion, Lowe syndrome should be suspected in males with congenital cataracts, developmental anomalies, and renal tubular dysfunction of the Fanconi type. The finding of a pathogenic DNA variation in OCRL1 gene can confirm the diagnosis. Early diagnosis and treatment of metabolic disturbances may delay the morbidity and mortality in this syndrome. 
